# Physico-Chemical Characterization and In Vitro Biological Evaluation of a Bionic Hydrogel Based on Hyaluronic Acid and l-Lysine for Medical Applications

**DOI:** 10.3390/pharmaceutics13081194

**Published:** 2021-08-03

**Authors:** Giuseppe Alonci, Roberto Mocchi, Sabrina Sommatis, Maria Chiara Capillo, Elsa Liga, Agata Janowska, Lidia Nachbaur, Nicola Zerbinati

**Affiliations:** 1 Qventis GmbH, 16761 Hennigsdorf, Germany; giuseppe.alonci@neauvia.com; 2 Matex Lab Switzerland SA, 1228 Plan-les-Ouates, Switzerland; 3 UB—CARE S.r.l.-Spin-off University of Pavia, 27100 Pavia, Italy; roberto.mocchi@ub-careitaly.it (R.M.); sabrina.sommatis@ub-careitaly.it (S.S.); mariachiara.capillo@ub-careitaly.it (M.C.C.); quality@ub-careitaly.it (E.L.); 4 Department of Dermatology, University of Pisa, 56121 Pisa, Italy; agata.janowska@unipi.it; 5 Department of Medicine and Surgery, University of Insubria, 21100 Varese, Italy

**Keywords:** regenerative medicine, hyaluronic acid, lysine, hydrogels, biocrosslinker, regeneration

## Abstract

Hyaluronic acid (HA) is an endogenous polysaccharide, whose hydrogels have been used in medical applications for decades. Here, we present a technology platform for stabilizing HA with a biocrosslinker, the amino acid l-Lysine, to manufacture bionic hydrogels for regenerative medicine. We synthetized bionic hydrogels with tailored composition with respect to HA concentration and degree of stabilization depending on the envisaged medical use. The structure of the hydrogels was assessed by microscopy and rheology, and the resorption behavior through enzymatic degradation with hyaluronidase. The biological compatibility was evaluated in vitro with human dermal fibroblast cell lines. HA bionic hydrogels stabilized with lysine show a 3D network structure, with a rheological profile that mimics biological matrixes, as a harmless biodegradable substrate for cell proliferation and regeneration and a promising candidate for wound healing and other medical applications.

## 1. Introduction

Hyaluronic acid (HA) is a structural building block widely present in the human body throughout the extracellular matrix, and vitreous, connective, epithelial and neural tissues [1,2]. Despite its structural simplicity and repetitiveness, a glycosaminoglycan consisting of *N*-acetylglucosamine–glucuronic acid, disaccharide units repeating in a linear fashion, HA plays a role in a broad spectrum of physiological processes, such as in morphogenesis and tissue organization, cell proliferation, differentiation and migration, among others [3,4,5]. 

HA is biocompatible, biodegradable, non-immunogenic and commercially available from non-animal sources with a high purity level at an affordable price, and for these reasons HA medical products have been used for decades in ophthalmology, joint health and facial aesthetics [6,7,8]. The biochemistry of HA is a field of intensive research due to the wide-ranging roles of this multitasking molecule in living systems and for developing further medical applications [3,9]. 

In living tissues, HA is stabilized by glycoproteins, together with collagen and elastin, building up the extracellular matrix 3D network and giving resilience to connective tissues and supporting cell proliferation, migration and, ultimately, regeneration. These characteristics make HA a promising candidate for applications in tissue engineering, wound healing and soft tissue regeneration [10,11,12,13,14].

In the human body, HA homeostasis is a dynamic process that involves a competition between synthesis and degradation. The exact HA degradation kinetics are dependent on the tissue, but the half-time of degradation normally ranges from hours to days [15]. The hyaluronidase enzymes HYAL 1 and HYAL 2 have been identified as the most active moieties in biodegradation of HA through hydrolysis of β-1,4-glycosidic linkages between N-acetyl-glucosamine (NAG) and D-glucuronic acid [16,17].

Therefore, for specific medical applications, e.g., restoration of age-related volume loss in facial soft tissue, it is important to stabilize HA through a crosslinking process, which turns the otherwise liquid viscous solution into a solid-like biohydrogel, that resembles the natural structure of HA in the body and allows one to tailor mechanical, biological and rheological properties (including injectability) and resorption time [18,19]. 

Available medical devices based on stabilized HA are usually crosslinked with 1,4-Butanediol-diglycil-ether (BDDE) [7,20]. 

Even if BDDE-crosslinked hyaluronic acid hydrogels are considered safe, BDDE itself is toxic and it is mandatory to purify the hydrogel to remove unreacted BDDE (residues must be below 2 ppm). Moreover, BDDE is a synthetic bridge with no potential biological activity to support cell proliferation.

A less common chemical modification of HA is esterification with benzyl alcohol, used to manufacture dressings and films for wound healing and advanced wound care (e.g., products of the HYAFF^®^ family) [21]. Other chemical routes to modify HA include, for example, the use of glutaraldehyde, divinyl sulfone, adipic acid dihydrazide derivatives, biscarbodiimide, acrylation and oxidation, or activation with N-(3-Dimethylaminopropyl)-N′-ethylcarbodiimide hydrochloride (EDC) and N-hydroxysuccinimmide (NHS). However, they all have very limited applications in the market because of safety concerns or because of complex and expensive manufacturing protocols [22,23,24,25,26].

Bionic design consists of integrating information from biological systems into the design and development of new products, in order to develop novel medical tools with good safety and efficacy profiles. We designed a bionic HA hydrogel stabilized with a physiologically occurring, bifunctional biomolecule, l-Lysine, which has the potential to support the repair of injuries or the age-related impaired structures or functions of living tissues.

Indeed, l-Lysine is an amino acid physiologically present in the body and is known for playing an important role in cell adhesion and collagen crosslinking [27,28]. However, pure l-Lysine is not reactive enough to react with the carboxylic or hydroxylic groups of HA. In this work, we present a new, straightforward platform technology for the crosslinking of hyaluronic acid with l-Lysine by using EDC/NHS, non-toxic coupling agents, that allow the preparation of homogeneous bionic hydrogels with a three-dimensional network, with tunable composition, HA concentration, degree of modification and rheological profile, tailored for specific uses in regenerative medicine. Further, we describe and present the results of physico-chemical characterization, degradation kinetics and in vitro biological evaluation.

## 2. Materials and Methods

### 2.1. Materials

Hyaluronic acid sodium salt (1 MDa, cosmetic grade) was supplied by HTL (Paris, France), N-hydroxysuccinimmide 98% was supplied by Alfa Aesar (Haverhill, MA, USA), while l-Lysine 98% and N-(3-Dimethylaminopropyl)-N′-ethylcarbodiimide hydrochloride (EDC) 98% were purchased from Sigma-Aldrich (St. Louis, MO, USA).

Hyaluronidase from bovine testes type I-S was purchased from Sigma-Aldrich (ref. H3506, 451 Units/mg).

All other chemicals were of the highest purity available.

Phosphate-buffered saline (PBS) solutions were prepared as described:

- PBS at pH = 6 ± 0.1 was obtained by dissolving in 1 L of water 4.5 g of NaCl, 1.10 g of Na_2_HPO_4_·2H_2_O and 6.88 g of NaH_2_PO4·2H_2_O and correcting the pH to the desired value with 1 M NaOH or 1 M HCl;
- PBS at pH = 7.4 was obtained by dissolving in 1 L of water 8.00 g of NaCl, 0.20 g of KCl, 1.78 g of Na_2_HPO_4_ · 2H_2_O and 0.24 g KH_2_PO4, and then adjusting the pH to 7.4 ± 0.1 with HCl 1 M or NaOH 1 M.

Dialysis was performed with a Spectra/Por^®^ 4 Dialysis Membrane, Standard RC Tubing, MWCO: 12–14 kD (Sigma-Aldrich).

### 2.2. HA Crosslinking

In a typical synthesis, 1 g of HA (2.6 mmol) is dissolved in 20 mL of a PBS solution at pH = 6. Then, 0.33 g of NHS (2.9 mmol) are dissolved in 1.25 g of water and added to the HA solution, followed by the addition of 1.50 g of EDC hydrochloride (7.8 mmol) dissolved in 2 g of water.

Afterwards, 1.00 g of a 25% solution of l-Lysine (1.7 mmol) in PBS at pH = 7.4 is added and the hydrogel obtained is left to react for 18 h. For the low crosslinked sample HA20L, 0.80 g of lysine solution are employed instead of 1.00 g.

The hydrogel is purified through dialysis for 5 days against PBS at pH = 7.4.

The initial (before dialysis) and final net weight are recorded and the HA concentration is adjusted as desired by adding PBS at pH = 7.4 (HA30 = 30 mg/mL; HA25 = 25 mg/mL; HA20 and HA20L = 20 mg/mL).

The hydrogel is autoclaved at 121 °C for 11 min and stored in sterile conditions.

### 2.3. Rheological Analysis

Oscillatory rheological analysis was performed with a DHR3 rheometer (TA Instruments, New Castle, DE, USA) equipped with a 35 mm parallel plate geometry, at a constant temperature of 37 °C to simulate the conditions in the human body.

Compression experiments were performed on a DHR3 rheometer (TA Instruments) equipped with rough 25 mm parallel plate geometry. For these tests, the gap was set to 1 mm. A frequency sweep from 0.5 to 5 Hz at 0.1% strain was performed. The gap was then set to 0.9 mm at 5 μm/s. Another frequency test was performed. The gap was then set to 1 mm again at the same speed. Another frequency test was performed. The cycle was then repeated 10 times in total.

To evaluate cohesivity and stretchability, extensional measures were carried on a Caber rheometer (Thermo Fisher, St. Louis, MA, USA) with 4 mm steel pads. The tests were performed over a distance of 10 mm in 9 s and the evolution of the normalized sample diameter with time was recorded. 

### 2.4. Microscopy

Optical images were obtained with a Keyence VHX-600 digital microscope (Osaka, Japan). SEM images were obtained in an environmental scanning electron microscope (FEI XL30 ESEM, FEI Technologies Inc., Fremont, CA, USA). The ESEM investigations were performed in high-vacuum mode of the microscope. The signal was processed with a signal mix of a backscatter electron detector (BSE detector) and a secondary electron detector (SE detector). 

The samples were frozen directly in liquid nitrogen and freeze dried for 24 h in a Christ GAMMA 1-16LSG. Afterwards, the samples were mounted on a sample holder and covered with gold.

### 2.5. Enzymatic Degradation Test

The enzymatic degradation of tested hydrogels was evaluated using a previously described protocol for the quantification of the released N-acetyl glucosamine (NAG) [29]. Hydrogels were weighed (0.2 g) and centrifuged in glass tubes at 1000× *g* using a refrigerated bench centrifuge (Megastar 600R, VWR, Milano, Italy). The hyaluronidase solution was prepared in a specific concentration (6080 U/mL) in isotonic phosphate–NaCl buffer at pH 7.4 and added onto the surface of the gels. After incubation, at different time points (1 h, 3 h, 6 h, 24 h, 48 h, 72 h, 120 h, 168 h), the enzymatic reaction was stopped by the addition of potassium tetraborate solution (0.8 M, pH 9.1), followed by vortexing and heating at 100 °C. NAG released in the solution was assayed according to the methods reported in the literature [30]. Briefly, Ehrlich’s reagent (Merck, Darmstadt, Germany) was diluted 1:10 in acetic acid (Merck) and added to the tubes; then, samples were vortexed and incubated for 20 min at 37 °C, to develop a violet color proportional to the NAG content in each sample. After centrifugation at 1000× *g* for 15 min, absorbance was recorded at a 585 nm wavelength with a microplate reader (Multiskan, Thermo Scientific, Waltham, MA, USA) against a blank prepared with only phosphate buffer and the Ehrlich’s reagent.

### 2.6. Data Analysis 

Data obtained from hyaluronidase sensitivity tests were analyzed by determining the NAG degradation percentage at each time point. The expected amount of NAG in each sample starting from the percentage of hyaluronic acid in each product was calculated. The obtained values were used as a reference to calculate the percentage of NAG released by hyaluronidase. The obtained data were plotted using the standard hyperbole equation (GraphPad Prism, San Diego, CA, USA):

$$y=\frac{ax}{\left(b-x\right)y}$$

Data points fitting to the model were evaluated by calculating the R_2_ for each sample analysis. Slope values between points 0–50% were calculated as well in order to determine the degradation rate for each product. t_½_ is defined as the time at which NAG degradation is half of the maximum; t 50% is defined as the time at which NAG degradation is equal to 50%. 

### 2.7. Cell Culture

Normal human dermal fibroblasts (NHDF-Ad—human dermal fibroblasts, adult, CC-2511 Lonza, Basel, Switzerland) were cultured in a complete medium constituted by high-glucose Dulbecco’s modified Eagle’s medium (DMEM, Biowest, Nuaillé, France) supplemented with 10% fetal bovine serum (FBS, Gibco-Fisher Scientific, Waltham, MA, USA) and 1% of l-glutamine (Capricorn Scientific, Ebsdorfergrund, Germany), penicillin (100 U/mL) and streptomycin (100 ug/mL) (Capricorn Scientific, Ebsdorfergrund, Germany), in conditions of complete sterility and maintained at 37 °C with a 5% carbon dioxide (CO_2_) atmosphere.

### 2.8. In Vitro Cell Biocompatibility

The evaluation of biocompatibility of human fibroblasts after seeding on HA25 hydrogel was assessed by optical microscopy and 3-(4,5-dimethylthiazol-2-yl)-2,5-diphenyltetrazolium bromide (MTT, Merck, Darmstadt, German) staining. Briefly, hydrogels (0.2 mL) were put inside a Petri dish (µ-Dish 35 mm high, Ibidi, Gräfelfing, Germany) and dermal fibroblasts were seeded on it at a density of 100,000 cells in complete culture medium. Then, after incubation at 37 °C, a morphological observation at different times (t = 0 h, 24 h, 48 h, 72 h and 144 h of incubation) was performed using an optical microscope (VisiScope IT415 PH, VWR part of Avantor, Milan, Italy). Afterwards, viable cells were evaluated using MTT staining. Briefly, after 144 h of cell incubation on hydrogels, a solution of MTT (1 mg/mL) was added at 37 °C for 2 h; samples were visualized under an optical microscope. 

### 2.9. Confocal Analysis of Cell Viability

Cell morphology and cell viability were also observed using a confocal microscope (Leica TCS SP8 STED 3X, Wetzlar, Germany). Normal human fibroblasts were seeded (100,000) on HA25 hydrogel, previously placed on a specific support (Ibidi, Gräfelfing, Germany), that led to a uniform distribution of the sample and better growth of cells on it. At different times, staining with the LIVE/DEAD™ Viability/Cytotoxicity Kit (Thermo Fisher Scientific) was performed. Briefly, a solution of calcein AM 2 µM and EthD-1 solution 4 µM was prepared in PBS (Sigma-Aldrich, St. Louis, MO, USA) and added to the samples; after incubation for 30 min, samples were analyzed by confocal microscopy.

## 3. Results

### 3.1. Rheological Characterization

The protocol allows for fine tuning of the rheological properties of the final hydrogel by playing with parameters such as molecular weight, initial HA concentration, final HA concentration and HA/EDC/NHS/lysine ratio. For example, Table 1 shows the rheological properties of different hydrogels prepared in different conditions. All the measures were performed after autoclaving the hydrogel at 121 °C for 11 min.

**Figure 1:**
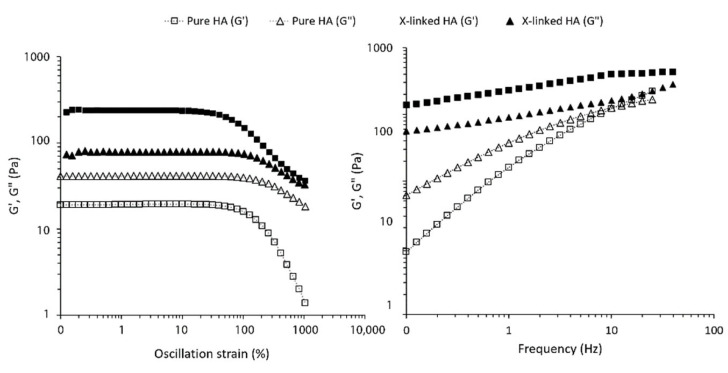
Amplitude sweep ((**left**), f = 1 Hz T = 37 °C) and frequency sweep ((**right**), γ = 1%, T = 37 °C) of a 2.5% pure HA solution vs. 2.5% HA-Lys hydrogel.

In Figure 1, we compare the rheological properties of a 2.5% solution of pristine HA with a 2.5% crosslinked hydrogel (HA25). Strain sweep and frequency sweep confirm that while pure HA mostly behaves as a viscous fluid (G′ < G″), HA crosslinked with l-Lysine in the same conditions behaves as an elastic gel (G′ > G″) and no crossover point was observed. This is a clear indication of the formation of a crosslinked network. 

We decided to explore the behavior of the hydrogel not only with oscillatory rheology, but also under repetitive axial stress to evaluate the response of the material under the conditions it may experience when used as a dermal filler or in intra-articular injections.

**Figure 2:**
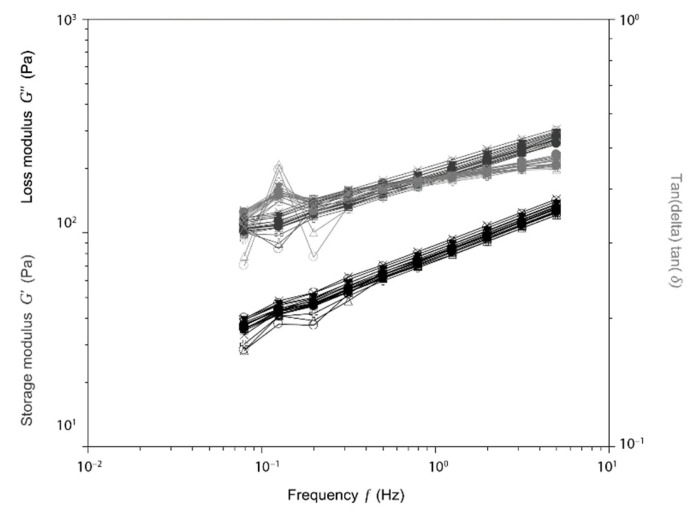
Compression cycles on HA30.

In Figure 2, we show the results of such experiments, in which repeated cycles of compression at different frequencies were performed on an l-Lysine crosslinked HA hydrogel with a 3.0% concentration. The HA–lysine hydrogels are able to withstand compression–elongation cycles at different frequencies without any significant change in their response, and thus no damage to their inner structure. Even at higher frequencies, the differences in the loss and storage modulus are negligible between each cycle. 

We also decided to perform extensional experiments that simulated the “finger test” commonly used for similar materials in the field of aesthetic medicine. In this test, each hydrogel was placed between the plates of a rheometer and the upper plate was raised in nine seconds by 10 mm. The experiment was performed on three HA-Lys hydrogels and for a standard commercial 28 mg/mL BDDE-crosslinked HA hydrogel as a reference (Renée Volume). Elasticity was calculated by interpolating the experimental data using the following mathematical model:

$$\frac{D\left(t\right)}{D_{0}}={\left(\frac{GD_{0}}{4\gamma }\right)}^{\frac{1}{3}}e^{\frac{t}{3\lambda_{c}}}$$

where *D*_0_ is the initial diameter of the filament (in meters); *G* the elastic modulus of the sample (in Pascal) and *λ_c_* the relaxation time (in seconds). Surface tension was fixed at 60 mN/m and density at 1033 kg/m^3^. In Table 2 we report the elastic modulus of the three hydrogels compared to a commercial dermal filler.

### 3.2. Imaging

We investigated the microscopic morphology of the hydrogel both through standard optical microscopy and through SEM microscopy.

**Figure 3:**
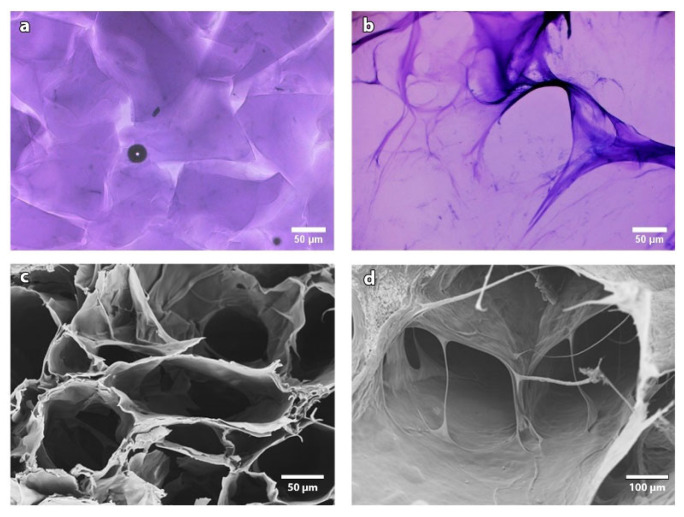
10× optical images of a (**a**) classical biphasic hydrogel and (**b**) sample HA30. SEM images of sample HA30 (**c**,**d**).

In Figure 3, we show the comparison at a 10× magnification of a classical biphasic hydrogel (Figure 3a) with HA-Lys hydrogel HA30 (Figure 3b), after staining of hyaluronic acid with toluidine blue. It is possible to observe that while classical monophasic HA hydrogels have a non-homogeneous structure, with crosslinked particles dispersed in un-crosslinked or poorly crosslinked gel, HA-Lys hydrogels have an isotropic, homogeneous structure. SEM analysis (Figure 3c,d) confirms that the material is composed of a crosslinked three-dimensional network of interconnected pores, with diameters ranging from 20 to 100 µm. This peculiar microscopic structure is extremely interesting, because it opens the possibility of employing the hydrogel as a scaffold for 3D cell culture, or as an ECM substitute in regenerative medicine.

### 3.3. Degradation

**Figure 4:**
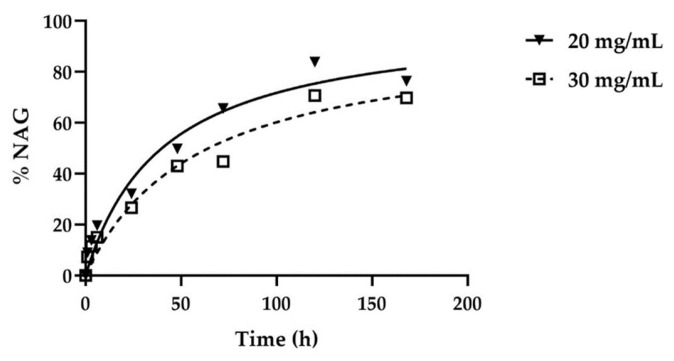
Trend of NAG released (%) in two different hydrogels containing different HA concentrations (20 or 30 mg/mL) after incubation with hyaluronidase at different timepoints (1/6/24/48/72/120/168 h). Data were plotted with the rectangular hyperbola equation as best-fitting curve model.

The data collected show that hydrogels are sensitive to bovine testes hyaluronidase degradation from the shortest timeframes analyzed. The best-fitting curve was obtained by plotting the absorbance mean of each sample, at each timepoint, with the rectangular hyperbola equation (Figure 4), obtaining the coefficients of determination as an index of data goodness of fit and the degradation parameters reported in Table 3. 

**Figure 5:**
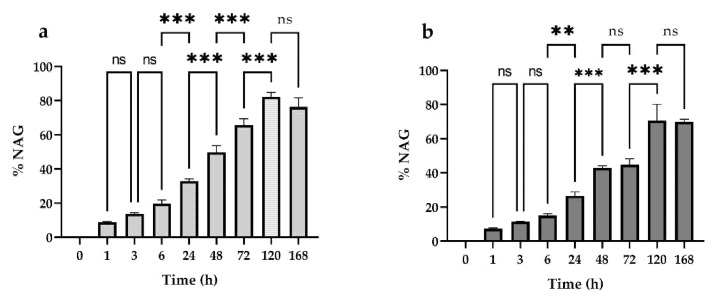
Statistical significance at each time point analyzed compared to the previous one. (**a**) HA 20 mg/mL; (**b**) HA 30 mg/mL. Values of *** *p* ≤ 0.001 or ** *p* ≤ 0.01 were considered statistically significant by one-way ANOVA statistical analysis followed by Šidák’s multiple comparisons as post-test (*n* = 2, replicates = 3).

The results show that HA20 presents the maximal percentage of NAG degradation (82.30%) and the quickest degradation rate (1.25%/h) with respect to HA30 (NAG degradation of 70.74% and degradation rate of 0.82%/h), demonstrating that the two degradation parameters are not dependent on the initial HA concentration, unlike the T_50%_ index. Indeed, a higher value of T_50%_ in HA30 demonstrates that the HA content is mostly associated with the initial hydrogel degradation [29]. As shown in Figure 5, the progressive time-dependent increase in the percentage of NAG release is statistically significant at all times analyzed except for the early ones (1/3/6 h) and the later ones (120/168 h), when it is possible that the release rate reached a plateau. In the case of HA30, the difference from 48/72 h is also not statistically significant.

### 3.4. In Vitro Evaluation of Hydrogel Biocompatibility

HA-based products are in direct and prolonged contact with human skin and mucous membranes and, consequently, they should show no or very low toxicity to skin cells or epithelia. In preliminary experiments, to evaluate the biocompatibility of the HA25 hydrogel, a normal human dermal fibroblast (NHDF) cell line was seeded on top of the hydrogel.

Subsequently, a morphological observation was performed using an optical microscope at different times (0/24/48/72 h and 144 h). 

**Figure 6:**
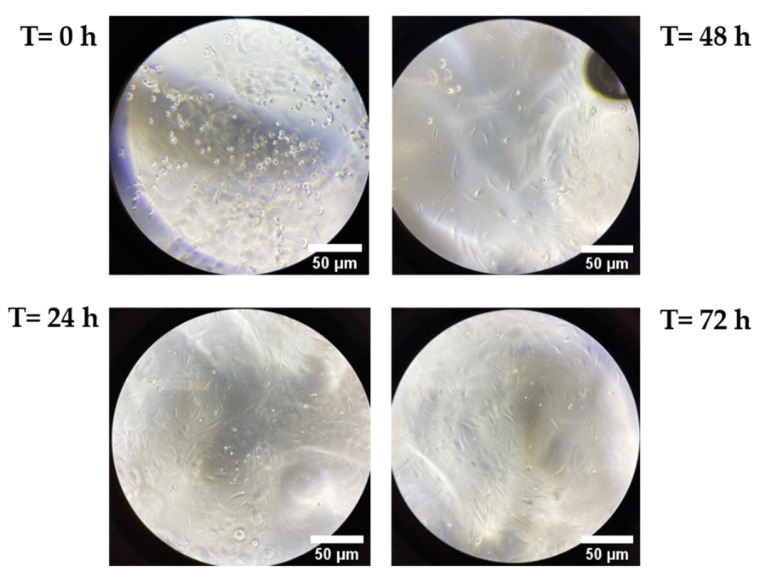
NHDF cells in the hydrogel observed under optical microscope (magnification 10×) after all analyzed times of incubation.

**Figure 7:**
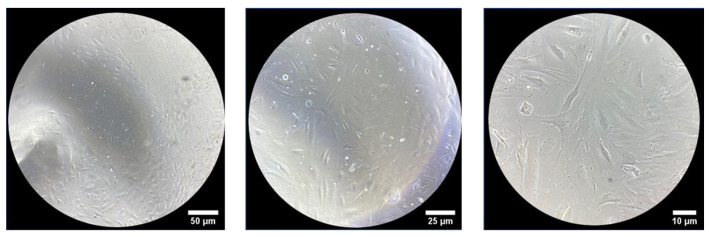
Representative images of NHDF cells in the hydrogel observed under optical microscope after 144 h of incubation. From left to right, magnification of 10×, 20× and 40×.

As shown in Figure 6, after 24 h, cells have a characteristic elongated shape that is maintained at all analyzed times up to 144 h of incubation with the hydrogels (Figure 7), suggesting the presence of viable cells. 

**Figure 8:**
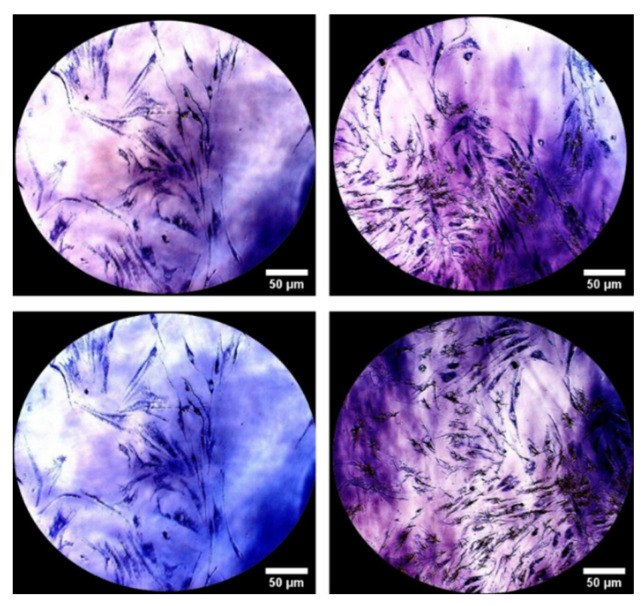
NHDF cells stained with MTT observed under optical microscope after 144 h of incubation (magnification 10×).

To further confirm the biocompatibility of the hydrogel, after 144 h of incubation, 3-(4,5-dimethylthiazol-2-yl)-2,5-diphenyl tetrazolium bromide (MTT) staining was performed to show mitochondrial activity; samples visualized by optical microscopy confirmed the presence of purple formazan crystals, the index of mitochondrial activity related to cell viability. These data confirmed an excellent compatibility of the hydrogel with NHDF cells, suggesting a good biocompatibility towards healthy skin (Figure 8). 

The ratio between live and dead cells was investigated through staining with the LIVE/DEAD™ Viability/Cytotoxicity Kit (Thermo Fisher Scientific, Waltham, MA, USA). 

**Figure 9:**
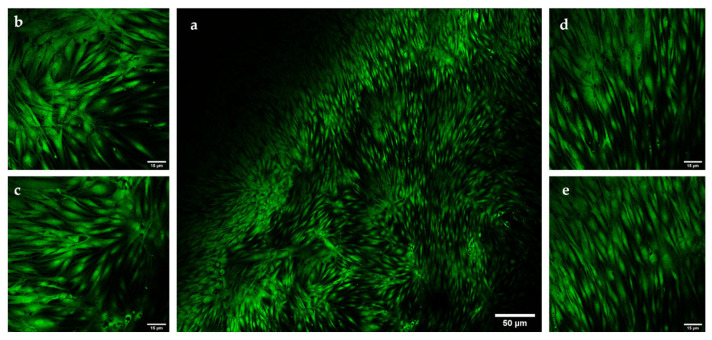
NHDF cells stained with LIVE/DEAD™ Kit observed under fluorescence confocal microscope after 144 h of incubation on sample HA25. The images on the sides (**b**–**e**) at magnification 63× are sections of the main picture (**a**), magnification 20×.

Figure 9 shows cells seeded on hydrogel and analyzed after 144 h of incubation with a fluorescence confocal microscope after staining. Green fluorescence indicated the presence of viable cells with a good morphology, demonstrating the excellent biocompatibility of the hydrogel.

## 4. Discussion

**Figure 10:**
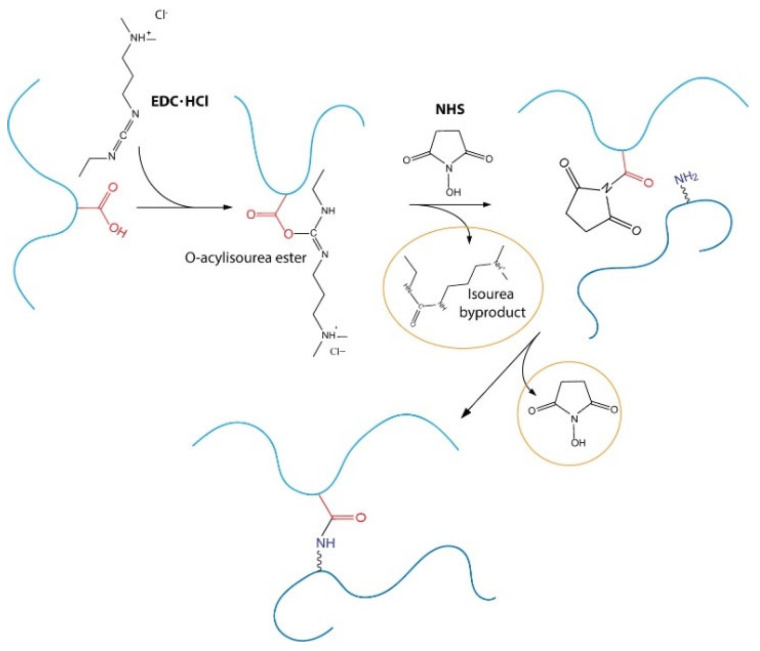
Reaction scheme of the crosslinking process.

l-lysine is not reactive enough to directly crosslink HA and a coupling strategy must be designed. We decided to employ the well-known N-(3-Dimethylaminopropyl)-N′-ethylcarbodiimide hydrochloride (EDC) and N-hydroxysuccinimmide (NHS) coupling approach, that has already been used for several years, for the crosslinking of amino acids. The mechanism of reaction is reported in Figure 10 and involves the activation of the carboxylic group by the EDC with the formation of an O-acylisourea ester, the formation of an NHS ester with the elimination of an isourea byproduct and, finally, coupling with the amino group that leads to the formation of an amide bond. 

The byproducts of the reaction are non-toxic and easy to remove through dialysis, while the amide bond is extremely stable in physiological conditions. 

Although similar approaches have already been attempted in the past by other authors [29,30,31], they were not successful in providing a simple, flexible and straightforward method. On the contrary, the studies often involved the use of lysine methyl ester during synthesis, followed by a second step of hydrolysis, which leads to the release of highly toxic methanol. This is necessary because, in the reported conditions, the carboxylic group in the alpha carbon of the l-Lysine would be activated and react to form an ester bond with HA, which is not stable in physiological conditions. Other methods also involve performing the reaction at higher pH and temperature, where HA is less stable, long purification processes that employ organic solvents or even destroying the 3D network to allow an easier handling of the gel. Although sterility is mandatory for the medical use of a hydrogel, the behavior of an HA hydrogel stabilized with l-Lysine after autoclaving has not been reported in the literature.

Considering that steam sterilization is a stressful process for HA-based materials, even when crosslinked, the physico-chemical characterization and biological evaluation of bionic HA hydrogel stabilized with l-Lysine formulations have been performed for autoclaved samples.

On the contrary, the synthetic strategy that we report is a simple, one pot synthesis and requires only one purification step, dialysis against PBS at physiological pH, and we show that the hydrogel is stable even after autoclaving. 

To optimize the crosslinking degree of the final product, several parameters must be tuned, such as temperature, order of addition of the reagents, concentration, reaction time and pH during the hydrogel preparation. The last parameter is of the utmost importance, not only because HA degradation is very sensitive to pH, but also because the EDC/NHS activation of the −COOH group works better at a pH between 5 and 6, while a basic pH greatly reduces the yield of the reaction by accelerating the hydrolysis of the intermediate. l-lysine coupling efficiency also depends on pH but, on the contrary, it is most favorable at a slightly basic pH, where the amino groups are unprotonated. As shown by the optical microscopy and SEM pictures reported in Figure 3, the hydrogel is a complex 3D microscopical structure made of interconnected pores that allows the diffusion of oxygen and nutrients inside the hydrogel and the proliferation of the cells.

Rheological studies were performed to demonstrate the efficiency of the crosslinking process, after autoclaving, and to characterize the mechanical behavior of the hydrogel under different conditions. A complete rheological characterization is crucial not only because it is necessary to understand the behavior of the hydrogel under stress during injection or after implantation, but the mechanical properties of the gel also influence cell growth and proliferation, especially for applications in tissue engineering, bone repair and osteo-articular fields [4,32,33,34].

The standard oscillatory protocols show that the crosslinking process is effective and all the samples have a loss module compatible with a gel-like material (i.e., G′ > G″ and tan δ < 1). The absolute value of G′ increases with the concentration of HA, as expected, and is lower for the less crosslinked HA20L sample in comparison to HA20.

The frequency sweep experiment (Figure 1) further demonstrates that the material has a gel-like behavior at all frequencies, and no crossover points are observed. 

To further understand the behavior of the hydrogel in more diverse conditions, we also performed compression and traction experiments. After several cycles of compressions (Figure 2), that mimicked what can happen when the hydrogel undergoes deformations by muscular activity or other physiological movements, the hydrogel still maintained its structural integrity even at a high frequency. The elastic modulus of the new hydrogels, reported in Table 2, are generally lower compared to a similar product in the market, making the hydrogel easier to stretch with a lower force. This is especially important if the hydrogel has to be injected into the dermis or a joint, because it means that it can better adapt to the surrounding tissues and comply with the natural stress produced by movement without causing any mechanical strain that may be uncomfortable for the patient. 

New HA-based formulations for medical applications require preclinical studies to evaluate their safety and stability, and to guarantee patient compliance for the following study phases. 

In our study, the biocompatibility of the lysine-crosslinked 2.5% HA hydrogel (HA25) was evaluated over a period of 144 h, after direct contact of the material with a normal human dermal fibroblast (NHDF) cell line. The morphological observation of the cells in direct contact with the hydrogel confirmed the characteristic elongated and healthy shape at each timepoint investigated (0–24–48–72–144 h). In order to collect further evidence of the NHDF cell line’s healthy state, MTT staining was performed (cell viability indicator) until 144 h of incubation. In this experiment, the yellow tetrazolium salt 3-(4,5-dimethylthiazol-2-yl)-2,5-diphenyltetrazolium bromide was reduced to the purple dye formazan by the metabolic activity of the living cells, in particular by NAD(P)H-dependent oxidoreductase enzymes located in the mitochondria.

At all the analyzed timepoints, the formazan crystals observed confirmed a good mitochondrial activity of the cells as an index of cell viability.

To further confirm the biocompatibility of the material, the ratio between live and dead cells was investigated through staining with a LIVE/DEAD™ Kit and confocal microscopic analysis. This experiment was based on the use of a mixture of two dyes, calcein-AM and ethidium homodimer-1, to evaluate the cells’ membrane integrity and the activity of esterases, a ubiquitous class of intracellular enzymes. The acetomethoxy derivative of calcein (calcein-AM) is a non-fluorescent dye that, upon hydrolysis of the acetomethoxy group induced by the activity of esterases, releases the green fluorescent dye calcein, with excitation and emission wavelengths of 495/515 nm. The second enzyme, ethidium homodimer-1, interacts with nucleic acids to form a red-emitting complex (excitation at 527 nm, emission at 624 nm). However, it can cross the healthy cellular membrane because of its positive charge, and thus can be used as an indicator of a damaged membrane. The confocal imaging after the LIVE/DEAD™ staining showed an imbalance in the signal in favor of green fluorescence (Figure 9), confirming again that the cells were in good condition and demonstrated the excellent biocompatibility of the hydrogel, and its safety and suitability for in vivo applications. 

The degradation kinetic of the hydrogel is another crucial parameter that must be evaluated, in order to understand its lifetime in the body after implantation (for example, in aesthetic medicine or tissue regeneration applications) but also to evaluate its reversibility upon minimally invasive injection of hyaluronidases. Even if one of the purposes of the crosslinking process is to increase the HA hydrogel’s resistance to endogenous hyaluronidases, the physicochemical features of the polymer must allow rapid degradation if inappropriate applications occur [35]. Therefore, understanding the susceptibility of this new material to hyaluronidase-mediated degradation is a valid way to complete its safety assessment and its chemical characterization. 

The sensitivity to bovine hyaluronidase type I-S was investigated by an in vitro assay under highly controlled conditions, as described in the previous sections. 

The results obtained show that the HA20 and HA30 hydrogels reach 82% and 70% maximum degradation, respectively, if placed in contact with 6080 U/mL of hyaluronidase enzyme for a period of 168 h (Figure 4). 

The HA concentration does not interfere with the final percentage of degradation, but it is related to the initial hydrogel degradation, while T_50%_ (time to reach 50% of the maximum degradation value) is higher for HA30 than HA20. The amount of N-acetyl-glucosamine (NAG) released at different contact timepoints with the hyaluronidase is statistically significant at each timepoint, except for the early ones (1/3/6 h) and the later ones (120–168 h), when a plateau is probably reached. 

HA30 does not show a significant increase in NAG release from 48–72 h. Altogether, the results obtained demonstrate a good susceptibility of the lysine-crosslinked HA hydrogel in respect to the exogenous hyaluronidases.

## 5. Conclusions

To meet the needs of a global aging population for quality of life, regenerative medicine is seeking biomaterials with a good safety profile for supporting/boosting the regeneration of impaired tissues or function, triggered by degeneration or injury. 

We developed a technology platform that can be scaled up to an industrial level, to synthesize a lysine-stabilized bionic hydrogel based on HA for regenerative medicine. 

We are able to manufacture hydrogels with customized composition and mechanical properties for the intended use, which can be applied as an injectable, film, topical or scaffold material. The bionic hydrogel mimics biological matrixes, offering an excellent substrate for cell growth and proliferation, with tunable resorption time. The applications in regenerative medicine range from dermal restoration of facial aesthetics, joint health, dentistry and women’s health to wound healing and tissue engineering. The preclinical tests are ongoing with excellent results to date. Clinical evaluation of safety and efficacy aims registration as class III medical devices for wound healing and other medical applications.

## 6. Patents

De Oliveira Barbosa Nachbaur, L. Alonci G. (2019) Method for the manufacture and use of a bionic hydrogel composition for medical applications (European Patent Application EP 3 666 278 A1, US Patent Application US20200179419A1).

## Figures and Tables

**Table 1:** HA concentration, elastic modulus (G′) and viscous modulus (G″) of four HA-Lys hydrogels. Sample HA20L was prepared with a smaller amount of crosslinking agents compared to HA20.

Sample	HA Concentration	G′ (Pa)	G″ (Pa)	Tan δ
HA30	30 mg/mL	425	102	0.37
HA25	25 mg/mL	247	82	0.39
HA20	20 mg/mL	110	55	0.55
HA20L	20 mg/mL	69	41	0.60

**Table 2:** Elasticity (elastic modulus) from extensional measures.

Sample	HA Concentration	Elasticity (G, kPa)
HA30	30 mg/mL	24
HA25	25 mg/mL	58
HA20	20 mg/mL	6
Commercial dermal filler	28 mg/mL	148

**Table 3:** Degradation parameters of analyzed hydrogels containing 20 or 30 mg/mL hyaluronic acid (HA). R^2^: determination factor for the data fitting evaluation; Deg Max (%): maximum percentage of HA degradation contained in hydrogels; T_1/2_ (h): time to reach the 50% of the Deg Max value; T_50%_ (h): time needed to reach 50% of the total HA degraded; Slope (%/h): degradation rate evaluated in 0–50% interval.

Product	HA Content	R^2^	Deg Max (%)	T_1/2_ (h)	T_50%_ (h)	Slope (%/h)
HA20	20 mg/mL	0.97	82.30 ± 2.46	39.90	40.09	1.25
HA30	30 mg/mL	0.96	70.74 ± 9.44	58.26	63.33	0.82

## Data Availability

All the data are available from the authors upon request.
